# Association between educational attainment and amyloid deposition across the spectrum from normal cognition to dementia: A meta-analysis

**DOI:** 10.1016/j.ibneur.2025.05.010

**Published:** 2025-06-12

**Authors:** Fatemeh Sedghi, Elaheh Foroughi, Farzad Sheikhzadeh, Mahya Ahmadpour Youshanlui, Ata Akhtari Kohnehshahri, Omid Karimzadeh, Sayedeh-Fatemeh Sadat-Madani, Hani Ghadri, Peyman Parhiz, Amirhesam Amirbeyk, Shaghayegh Afshari, Yegane Ebrahimnia, Mahsa Soleimanzadeh, Mahsa Asadi Anar, Parviz Aghaei Borzabad, Niloofar Deravi

**Affiliations:** a Department of Health Education & Health Promotion, Health Faculty, Mashhad University of Medical Sciences, Mashhad, Iran; b Student Research Committee, Isfahan University of Medical Sciences, Isfahan, Iran; c School of medicine, Iran University of Medical Sciences, Tehran, Iran; d Immunology research center, Tabriz university of medical science, Tabriz, Iran; e Student Research Committee, Faculty of medicine, Tabriz Medical Sciences, Islamic Azad University, Tabriz, Iran; f Student research committee, Paramedical School, Zahedan University of Medical Sciences, Zahedan, Iran; g Medical Doctor, School of Medicine, Isfahan University of Medical Sciences, Isfahan, Iran; h Student research committee, School of Medicine, Iran University of Medical Sciences, Tehran, Iran; i Student research committee, Zahedan Medical Sciences Branch, Islamic Azad University, Zahedan, Iran; j Student Research Committee, School of Medicine, Mashhad University of Medical Sciences, Mashhad, Iran; k Student Research Committee, School of Medicine, Shahroud University of Medical Sciences, Shahroud, Iran; l Student Research Committee, Shahid Beheshti University of Medical Sciences, Tehran, Iran; m Assistant Professor, Department of Social Determinant of Health Research Center, Yasuj University of Medical Sciences, Yasuj, Iran

**Keywords:** Alzheimer's disease, Amyloid Beta, Mild Cognitive Impairment, Educational, Education

## Abstract

**Background & Aim:**

Educational attainment has been proposed as a critical factor influencing cognitive resilience in the face of neurodegenerative diseases. However, its relationship with amyloid deposition across different stages of cognitive decline remains unclear. This systematic review and meta-analysis aimed to investigate the correlation between educational background and amyloid accumulation in individuals ranging from cognitively normal to those diagnosed with Alzheimer’s disease (AD) or mild cognitive impairment (MCI).

**Methods:**

A comprehensive literature search was conducted in January 2025 across PubMed, Scopus, and Google Scholar to identify observational studies examining the association between educational attainment and amyloid deposition. Studies were included if they reported relevant data on this relationship; reviews, interventional studies, and those with insufficient outcome reporting were excluded. Data were analyzed using RStudio (version 4.3.1), employing random-effects or fixed-effects models based on the degree of heterogeneity. Effect sizes were expressed as Pearson’s correlation coefficients (r).

**Results:**

Out of 6988 initially identified records, four cohort studies met the inclusion criteria, comprising a total of 288 participants from Canada and Japan. Meta-analysis revealed a significant positive correlation between educational attainment and amyloid deposition among individuals with MCI (r = 0.34, 95 % CI [0.05, 0.63]), although substantial heterogeneity was observed (I² = 86 %, p < 0.01). In contrast, an inverse association was found in patients with AD (r = -0.16, 95 % CI [-0.28, −0.03]), with minimal heterogeneity (I² = 0.0 %, p = 0.8101). Funnel plot analyses indicated no significant evidence of publication bias.

**Conclusion:**

These findings suggest a stage-dependent relationship between educational attainment and amyloid accumulation: higher education is associated with greater amyloid deposition in individuals with MCI, but with reduced amyloid burden in those with AD. This pattern supports the cognitive reserve hypothesis, which posits that education may bolster compensatory neural mechanisms, delaying the clinical onset of dementia symptoms. However, the presence of substantial heterogeneity and the limited sample size call for further longitudinal research to elucidate underlying causal pathways and inform targeted strategies for dementia prevention.

## Introduction

Subcortical vascular dementia (SVaD) and Alzheimer’s disease dementia (ADD) are among the most common forms of dementia, each with distinct pathological features—SVaD is typically marked by widespread white matter hyperintensities (WMH), while ADD is primarily associated with amyloid-beta (Aβ) plaque accumulation. Emerging evidence suggests that individuals with lower educational attainment may be at increased risk for both types of dementia (Hwangbo et al., 2022). One prevailing hypothesis is that lower educational attainment is associated with socioeconomic disparities, which may lead to poorer health behaviors, increased exposure to environmental risk factors, and inadequately managed vascular conditions—all of which can compound the risk for dementia (Livingston et al., 2020, Norton et al., 2014). Research on resilience mechanisms has shown that individuals with higher educational attainment who are diagnosed with mild cognitive impairment (MCI) or dementia tend to exhibit greater levels of amyloid and tau pathology than those with lower education, despite having comparable clinical symptoms. This suggests that higher education may lessen symptom severity or postpone the emergence of noticeable cognitive deficits. Recently, there has been growing interest in exploring the protective role of education during the preclinical stages of AD, particularly in individuals experiencing subjective cognitive decline (SCD) (Hönig et al., 2024).

The association between educational attainment and reduced Alzheimer’s disease (AD) risk is widely attributed to the concept of cognitive reserve. This theory posits that the brain can compensate for neurodegenerative changes through more efficient, adaptable, and complex neural networks. While cognitive reserve is often linked to lifelong intellectual engagement and mentally stimulating activities, it is fundamentally rooted in neurobiological mechanisms such as increased synaptic density, enhanced neural network efficiency, and compensatory functional reorganization (Meng and D’arcy, 2012). Neuroimaging studies support this framework, indicating that individuals with higher levels of education may demonstrate greater neural efficiency and resilience, enabling them to preserve cognitive function despite significant accumulation of Alzheimer’s pathology (Del Ser et al., 1999).

Epidemiological studies have consistently found that individuals with higher levels of education are at a lower risk of developing Alzheimer’s disease (Hönig et al., 2024). This protective association is often explained by the concept of cognitive reserve, which refers to the brain’s ability to cope with damage through more efficient or flexible neural processes. Essentially, people with more education may have a stronger cognitive “buffer,” allowing them to function normally even when early brain changes of Alzheimer’s disease—such as amyloid-beta (Aβ) accumulation—begin to appear (Meng and D’arcy, 2012). However, neuropathological evidence suggests that higher education does not actually prevent the formation of Aβ plaques in the brain. Instead, what seems to happen is that individuals with more education can tolerate higher levels of amyloid pathology before showing noticeable cognitive decline. In other words, while the underlying disease process may be progressing, the symptoms remain hidden for longer due to the brain’s enhanced ability to compensate (Del Ser et al., 1999).

Neuroimaging studies support this idea. They show that highly educated individuals diagnosed with Alzheimer’s disease often have greater Aβ deposition compared to those with less education who are at the same stage of cognitive impairment (Mortimer et al., 2005). This apparent paradox reinforces the cognitive reserve hypothesis, proposing that complex neural networks help buffer against the effects of neuropathology. In addition to education, other factors—such as lifelong cognitive stimulation through work, hobbies, and intellectual activities—may also contribute to structural brain resilience. Recent studies suggest that engaging in mentally stimulating activities, particularly during early and midlife, may promote healthier brain structures and networks. This resilience might influence how amyloid pathology develops or is managed in individuals who are still cognitively healthy, possibly affecting the early, preclinical stages of Alzheimer’s disease (Roe et al., 2007).

Despite these insights, significant gaps remain in the literature. Many studies either focus on clinical progression or neuroimaging markers, but few integrate these elements to explore how educational attainment affects amyloid deposition across the full continuum of cognitive functioning—from normal cognition to dementia. To date, no systematic review has comprehensively synthesized the available neuroimaging and clinical evidence to clarify the relationship between education and Aβ burden.

This meta-analysis aims to address this critical gap by evaluating the association between educational attainment and Aβ deposition across varying cognitive states. By integrating data from both neuroimaging and clinical studies, we seek to enhance our understanding of the role education plays in modifying Alzheimer’s disease risk and progression.

## Material and method

This study investigates the relationship between educational attainment and amyloid deposition across the cognitive spectrum, from normal cognitive function to dementia. To ensure transparency and adherence to best practices, the research protocol was preregistered on the Open Science Framework (OSF) (https://doi.org/10.17605/OSF.IO/XSR6M). The study followed a rigorous methodology aligned with the Preferred Reporting Items for Systematic Reviews and Meta-Analyses (PRISMA) guidelines, encompassing systematic literature search, study selection, quality assessment, and statistical analysis.

### Search strategy

A comprehensive literature search was conducted in January 2025 across PubMed, Scopus, and Google Scholar to identify relevant studies published up until 2023. Advanced search strategies, including Boolean operators and database-specific tags, were utilized. The complete search strategy for each database is detailed in Table 1. After duplicate removal, two independent researchers screened the titles and abstracts of the retrieved studies, selecting those that met the inclusion criteria for full-text review. Disagreements were resolved through consensus (Table 2).

**Table 1:** Search strategies for PubMed/MEDLINE, scopus, and google scholar databases.

**Database**	**Search Terms**	**Results** **Search date:** (January 29,2025**)**
**PubMed**	(education[tiab] OR educate[tiab] OR educational[tiab]) AND ("Amyloid"[Mesh] OR "Amyloid"[tiab]) AND ("Dementia"[tiab] OR "Alzheimer Disease"[tiab] OR "Dementias, Vascular"[tiab] OR "Vascular Dementia"[tiab] OR "vascular dementias"[tiab] OR "Lewy Body Disease"[tiab] OR "Lewy Body Dementia"[tiab] OR "Senile Dementia"[tiab] OR "Senile Dementias"[tiab] OR "Presenile Dementia"[tiab] OR "Presenile Dementias"[tiab] OR "Alzheimer's Disease"[tiab] OR "Alzheimer Dementia"[tiab] OR "Alzheimer's Dementia"[tiab] OR "Alzheimer Dementias"[tiab] OR "Alzheimer's Dementias"[tiab] OR "cognitive impairment"[tiab] OR "cognitive decline"[tiab])	1019
**Scopus**	(TITLE-ABS-KEY ((dementia* OR Alzheimer* OR “Lewy Body Disease” OR “cognitive impairment” OR “cognitive decline”))) AND (TITLE-ABS-KEY (amyloid*)) AND (TITLE-ABS-KEY (education* OR educate* OR educational*))	1845
**Google Scholar**	education| educate| educational Amyloid Dementia Alzheimer | "Alzheimer Disease"|"Lewy Body Disease"|"Lewy Body Dementia"|"Senile Dementia"|"cognitive impairment"|"cognitive decline"	4840
**ALL**		7704

**Table 2:** Description of included studies.

Author (Year)	Country	Study Design	Sample Size (MCI / AD)	Age (Mean ± SD)	Male (n%)	Educational Level (Years + Stratified Categories)	Amyloid Measurement Modality	Tracer & Region of Interest	Aβ Positivity Threshold	Cognitive Status	APOE ε4 Adjusted	Psychiatric/Neurological Inclusion	Exclusion Sensitivity	Reported Correlation
Arenaza-Urquijo et al. (2017) (Hwangbo et al., 2022)	France	Cross-sectional	140 (44 / 23)	73.7 ± 6.9 for MCI and 69.5 ± 9.3 for AD	56.8 for MCI and 47.82 for AD	6–20 years; not stratified; no group difference	PET	Florbetapir; SUVR in global cortex excluding cerebellum, occipital, sensorimotor, hippocampus, amygdala, thalamus	SUVR Index; exact cutoff not specified	CN, MCI, AD	Yes (included in regression models)	CN excluded for psychiatric/neurological disorders; not reported for MCI/AD	No exclusion sensitivity reported	CN: r = −0.3; MCI: r = 0.4; AD: r = 0.003
Yasuno (2020) (Livingston et al., 2020)	USA	Cross-sectional	26 (24/2)	73.3 ± 5.1	61.54	Mean 16.3 ± 2.8 years; stratified: Lower (12–16 yrs, HI 4–6) vs. Higher (17–20 yrs, HI = 7)	PET	AV−45 (florbetapir) – cortical regions (frontal, cingulate, parietal, temporal); AV−1451 (flortaucipir) – Braak stages 1–6		MCI and mild AD	Yes (as covariate in partial correlation models)	Excluded dementia, depression, behavioral medication	Excluded individuals on antidementia, antidepressant, and behavioral meds; only included subjects with complete neuropsych and imaging data	Significant positive correlation between education and tau in Braak 1/2 (r = 0.26, *p* = 0.003); no correlation with amyloid or in Braak 3/4 or 5/6; no correlation with SES/occupation
Hoenig et al. (2022) (Norton et al., 2014)	Multiple European countries (8 sites)	Cross-sectional / Propensity Score Matched Analysis	227 MCI / 157 AD			Median split; Higher vs. Lower Education based on years (exact years not given); median values per site	PET	Flutemetamol and Florbetaben; Global Centiloid (CL) values		SCD+ , MCI, AD	Yes (controlled in analysis)	SCD+ , MCI, and AD patients included; exclusion of individuals at median education	Matching for age, sex, MMSE; Partial correlation used as sensitivity analysis	MCI: r = .153, p = .022 (positive); SCD+ : r = −.159, p = .027 (negative); AD: No correlation
Binette et al. (2021) (Hönig et al., 2024)	Canada / Multinational (DIAN)	Cross-sectional observational study	232 CU (115 PREVENT-AD / 117 DIAN)	PREVENT-AD: 67.6 ± 5.0DIAN: 34.6 ± 9.4	PREVENT-AD: 25 % DIAN: 45 %	PREVENT-AD: 15.0 ± 3.2 (Mortimer et al., 2005, Roe et al., 2007, Sharp and Gatz, 2011, Caamaño-Isorna et al., 2006, Brayne et al., 2010, Yasuno et al., 2020, Ingber et al., 2016, Hoenig et al., 2017, Scarmeas et al., 2006, Stern et al., 1995, Valenzuela and Sachdev, 2006, Barulli and Stern, 2013, Hall et al., 2009, Yu et al., 2012, Muniz-Terrera et al., 2013, Stern, 2012)DIAN: 15.2 ± 3.0 (Caamaño-Isorna et al., 2006, Brayne et al., 2010, Yasuno et al., 2020, Ingber et al., 2016, Hoenig et al., 2017, Scarmeas et al., 2006, Stern et al., 1995, Valenzuela and Sachdev, 2006, Barulli and Stern, 2013, Hall et al., 2009, Yu et al., 2012, Muniz-Terrera et al., 2013, Stern, 2012, Arenaza-Urquijo et al., 2017, Binette et al., 2021)	PET (Aβ in both; Tau in PREVENT-AD only)	PREVENT-AD: [18 F]NAV4694 (global Aβ SUVR)DIAN: [11 C]PIB (global Aβ SUVR)ROI: Lateral/medial prefrontal, parietal, temporal, cingulate cortices		Cognitively unimpaired (CDR = 0, MoCA >25)	Yes	Excluded major neurological & psychiatric illness	Moderate	PREVENT-AD: Neuroticism positively correlated with Aβ (R =.21, p = .02); Lifetime cognitive activity & apathy correlated with Tau (R =.21–.34, all p < .05)DIAN: Education inversely correlated with Aβ (R = –.19, p = .04)

### Inclusion and exclusion criteria

To ensure methodological rigor and reproducibility, we applied explicit inclusion and exclusion criteria in selecting studies for this meta-analysis.

Studies were eligible for inclusion in this meta-analysis if they met the following criteria:

• Study Design: Only observational studies (cross-sectional, cohort, or case-control) were included to maintain consistency in study design and ensure that causal inference was not assumed.
• Population: Studies had to assess the association between educational attainment and amyloid-beta (Aβ) deposition in human populations, including cognitively normal individuals, individuals with mild cognitive impairment (MCI), and those with Alzheimer’s disease (AD).
• Outcome Reporting: Only studies that reported a Pearson correlation coefficient (r) between education and Aβ deposition, or provided sufficient statistical data to calculate it, were considered eligible.
• Measurement of Aβ Deposition: Included studies were required to use in vivo imaging techniques (e.g., PET scans with amyloid-specific tracers) or cerebrospinal fluid (CSF) biomarkers to quantitatively assess Aβ deposition.
• Diagnostic Criteria: Studies were required to include clear clinical definitions of MCI and AD, based on established diagnostic standards such as the Petersen criteria, the National Institute on Aging–Alzheimer’s Association (NIA-AA) guidelines, or the Diagnostic and Statistical Manual of Mental Disorders, Fifth Edition (DSM-5). Given that MCI often progresses to AD, studies had to report outcomes separately for MCI and AD groups to minimize diagnostic overlap.
• Publication Quality: Only articles published in peer-reviewed journals and written in English were included to ensure scientific rigor and accessibility.

Exclusion Criteria:

• Study Type: Interventional trials, reviews, meta-analyses, case reports, case series, editorials, letters, posters, and conference abstracts were excluded due to insufficient methodological and statistical detail for quantitative synthesis.
• Data Availability: Studies that did not report or allow the extraction of a Pearson correlation coefficient between educational attainment and Aβ deposition were excluded.
• Confounding Conditions: Studies that included participants with co-occurring neurological (e.g., Parkinson’s disease, stroke, traumatic brain injury) or psychiatric disorders (e.g., major depressive disorder, schizophrenia) were excluded to reduce confounding effects on amyloid deposition and cognitive performance.
• Covariate Adjustment: Articles were excluded if they failed to control for major confounding variables, including age, sex, and APOE ε4 genotype, a well-established genetic risk factor for Aβ accumulation. Preference was given to studies that performed multivariable statistical adjustments, including genetic and biological modulators of Aβ pathology.

### Quality assessment and data extraction

The methodological quality of the included studies was assessed using the Joanna Briggs Institute (JBI) critical appraisal checklist for observational studies (https://jbi.global/critical-appraisal-tools). Two independent evaluators assessed the full texts, and any discrepancies were resolved through discussion. We judged studies that received a score of one-half of the total score to be at low risk of bias, and studies that scored less than one-half of the total score to be at high risk and we eliminated them. Key study characteristics, including Author (Year), Country, Study DesignSample Size (MCI / AD), Age (Mean ± SD), Male (n%), Educational Level (Years + Stratified Categories), Amyloid Measurement Modality, Tracer & Region of InterestAβ, Positivity Threshold, Cognitive Status, APOE ε4 Adjusted, Psychiatric/Neurological Inclusion, Exclusion Sensitivity, and Reported Correlation, were extracted using a standardized data collection form. Two independent researchers performed data extraction to ensure accuracy.

### Educational attainment assessment

Educational attainment was operationalized based on the information reported in each study. In most cases, this was defined as total years of education; however, some studies may have relied on self-reported academic degrees or educational categories. When available, years of education were used as a continuous variable or stratified into groups (e.g., high vs. low education) based on median splits. Given the cross-cultural nature of the included studies, we note that the interpretation of "years of education" may differ across countries due to variations in education systems.

### Statistical analysis

The extracted data were organized into a standardized spreadsheet using Excel. All statistical analyses were conducted using RStudio (version 4.3.1) and the meta package. To ensure consistency, reliability estimates were standardized using Fisher's Z-transformation and reported as Pearson's correlation coefficients. Cochran's Q test was employed to assess the heterogeneity of effect sizes, while the I² statistic was used to quantify the degree of heterogeneity. All statistical tests were conducted at a significance level of 0.05. Funnel plots were visually inspected to assess potential publication bias.

## Result

### Literature search

**Fig. 1:**
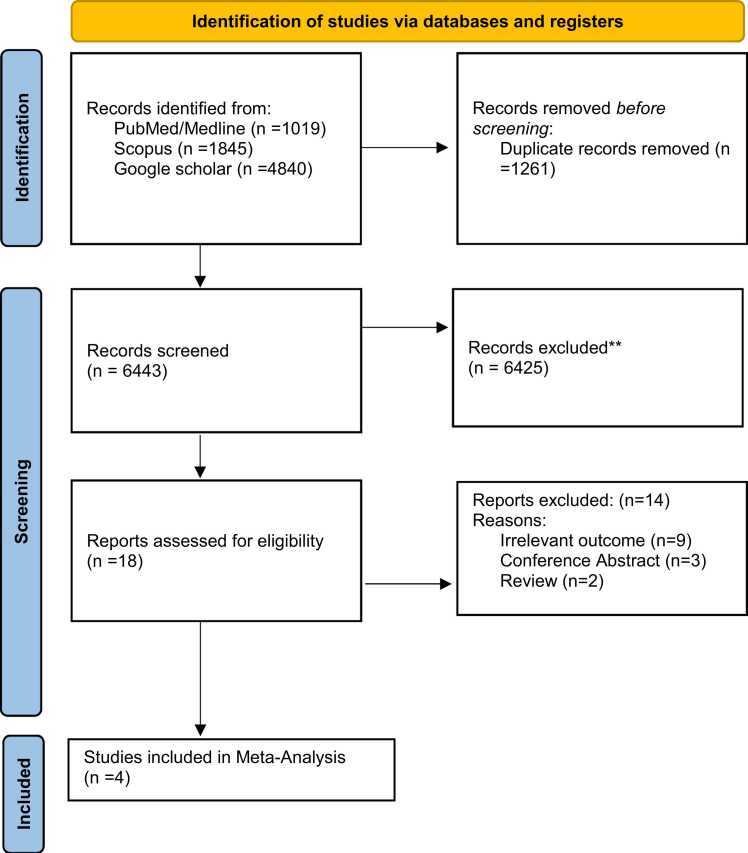
PRISMA DIAGRAM.

A total of 6988 articles were retrieved using the search strategy outlined in Table 1. After duplicates were removed, 5727 articles remained. Following primary and secondary screening, 18 articles were assessed for eligibility. Of these, 12 were excluded due to poor quality or failure to meet the specified outcomes. Ultimately, 4 studies were included in the analysis regarding the impact of health education on diabetic retinopathy screening (Fig. 1).

### Characteristics of eligible studies

The systematic review included 288 patients diagnosed with Alzheimer's disease (AD) or Mild Cognitive Impairment (MCI). The studies assessing the relationship between amyloid-beta (Aβ) deposition and educational attainment included patient populations ranging from 2 to 117 participants for AD and from 44 to 227 participants for MCI. Three cohort studies were included in the meta-analysis: two conducted in Canada and one in Japan. These studies were published between 2017 and 2022. Key characteristics of the included studies, such as study design, sample size, and setting, are summarized in Table 1. Quality assessment, conducted using the Joanna Briggs Institute (JBI) checklist, indicated that all included studies were of good quality.

### Relationship between educational attainment and amyloid deposition in MCI

**Fig. 2:**
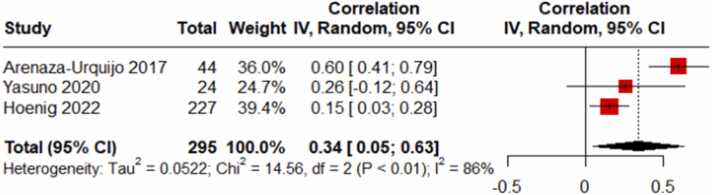
Forest plot of the Meta-Analyses focusing on the correlation between educational attainment and amyloid deposition in MCI.

**Fig. 3:**
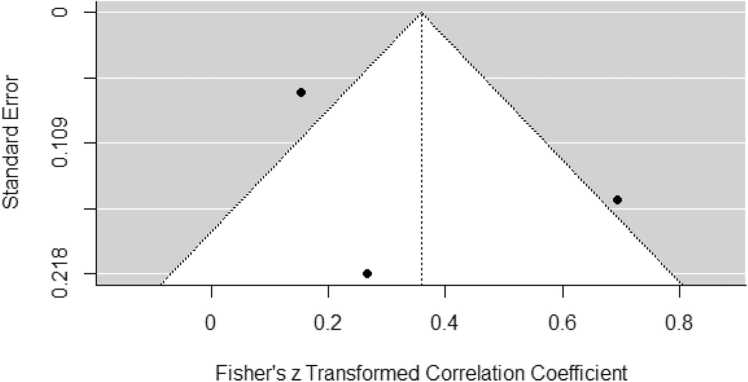
Funnel plot of the Meta-Analyses focusing on the correlation between educational attainment and amyloid deposition in MCI.

Initial analyses revealed significant heterogeneity in effect sizes across studies (I² = 86 %, p < 0.01). This indicated the need for a random-effects model. A significant positive correlation was found between educational attainment and amyloid deposition (r = 0.34, 95 % CI [0.05, 0.63]) (Fig. 2). This relationship is visually represented in Fig. 1. To assess potential publication bias, funnel plots (Fig. 3) were examined and it indicate no significant publication bias.

### Relationship between educational attainment and amyloid deposition in AD

**Fig. 4:**
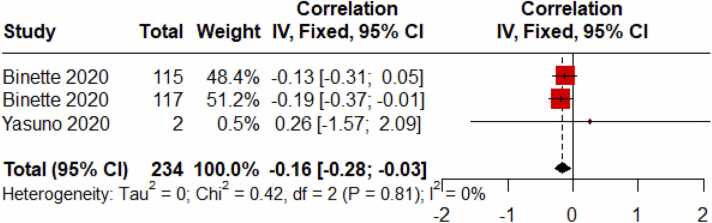
Forest plot of the Meta-Analyses focusing on the correlation between educational attainment and amyloid deposition in AD.

**Fig. 5:**
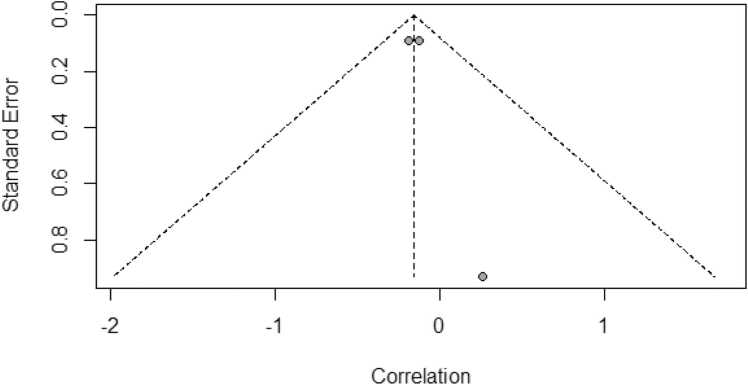
Funnel plot of the Meta-Analyses focusing on the correlation between educational attainment and amyloid deposition in AD.

Initial analyses indicated low heterogeneity in effect sizes across studies (I² = 0.0 %, p = 0.8101), suggesting the appropriateness of a fixed-effect model. A significant negative correlation was found between educational attainment and amyloid deposition (r = -0.16, 95 % CI [-0.28, −0.03]). This relationship is visualized in Fig. 4. Funnel plot analysis (Fig. 5) revealed no evidence of publication bias.

## Discussion

This meta-analysis, comprising four studies with a total of 288 participants, offers valuable insights into the complex relationship between educational attainment and amyloid-beta accumulation across various stages of cognitive health, ranging from normal cognition to dementia. Our findings reveal a notable distinction in amyloid-beta deposition patterns between individuals with Mild Cognitive Impairment (MCI) and those with Alzheimer's Disease (AD). Specifically, in individuals with MCI, higher educational attainment was associated with greater amyloid-beta accumulation compared to their less educated counterparts (r = 0.34, 95 % CI [0.05, 0.63]). In contrast, in individuals diagnosed with AD, the relationship was reversed, with higher educational attainment linked to lower amyloid-beta accumulation (r = -0.16, 95 % CI [-0.28, −0.03]).

These divergent findings align with the cognitive reserve hypothesis, which posits that individuals with higher educational levels may have enhanced neural networks, enabling them to withstand greater neuropathological burden before exhibiting clinical symptoms of cognitive decline. This hypothesis is further supported by previous research, such as Mortimer et al.'s study, which found no significant relationship between academic achievement and pathological Alzheimer's Disease criteria in a cohort of 60 nuns, although the study did not account for vascular contributions to dementia pathology (Mortimer et al., 2005). Similarly, Roe et al.'s analysis of data from the National Alzheimer’s Disease Coordinating Center (NACC) identified a link between education and dementia, although they did not replicate the specific interaction between education, pathology, and cognition observed in other cohorts (Roe et al., 2007).

Recent findings from individuals with subjective cognitive decline plus (SCD+) further highlight this complex interplay. In preclinical stages of AD, individuals with higher education reported heightened awareness of subtle cognitive changes, potentially due to increased sensitivity to the effects of early, subthreshold pathology. This suggests that higher education may enhance perceptual awareness of early dysfunction. Conversely, in clinical stages of AD, higher education appears to confer greater cognitive resilience against the effects of more substantial amyloid burden, likely through compensatory neural mechanisms. These findings support the notion that education has stage-specific effects—facilitating early detection through subjective awareness in preclinical stages, and mitigating symptomatic severity through resilience in later stages. Moreover, access to healthcare, proactive health-seeking behavior, and premorbid intelligence may contribute to earlier recognition and delayed progression, though longitudinal studies are required to confirm this hypothesis (Hönig et al., 2024).

Although lower educational attainment is commonly viewed as a risk factor for clinically diagnosed Alzheimer's disease dementia (ADD), Hwangbo et al.’s (Hwangbo et al., 2022) findings did not reveal a significant association between lower education levels and Aβ-positive ADD. This result contrasts with earlier studies (Sharp and Gatz, 2011, Caamaño-Isorna et al., 2006), and the discrepancy may be attributed to differences in study populations. Specifically, their analysis focused on biologically confirmed Aβ+ ADD cases, whereas many earlier studies relied on clinical diagnoses alone. This divergence in diagnostic criteria may help explain the inconsistencies.

In the study by Brayne et al (Brayne et al., 2010)., the relationship between education, neuropathology, and dementia was examined using data from the Epidemiological Clinical Pathology Studies in Europe (EClipSE), which included 872 participants, 44 % of whom were dementia-free. While the study did not find a significant overall association between education and neuropathological measures such as brain weight, neurological markers, or cardiovascular disease (CVD), a more nuanced pattern emerged when considering dementia risk. Specifically, education was associated with a reduced risk of dementia in individuals with lower brain weight, an effect not observed in those with higher brain weight. A similar pattern was seen with respect to the Braak stage of Alzheimer’s pathology: education was linked to a lower likelihood of dementia among individuals with low Braak stages, but this protective effect was absent in those with high Braak stages. These findings were consistent with those from the National Alzheimer's Disease Coordinating Center (NACC), reinforcing the idea that education's protective role may be limited to earlier stages of pathology.

A complementary study by Fumihiko Yasuno et al (Yasuno et al., 2020). further explored the relationship between education and Alzheimer’s pathology, focusing on tau accumulation in Braak stage 1/2 brain regions. The researchers found a significant positive correlation between higher education levels and greater tau pathology in these early-stage regions. Importantly, this relationship persisted after adjusting for potential confounders, including age, gender, clinical status, and ADAS-cog score. Despite the increased tau burden, individuals with higher education demonstrated better cognitive performance, suggesting a compensatory role for education. Notably, the impact of tau accumulation on cognitive performance was more pronounced among those with lower educational attainment, supporting the theory that education may buffer the cognitive effects of neuropathological damage in the early stages of Alzheimer’s disease (AD).

This compensatory effect of education is supported by findings from Ingber et al (Ingber et al., 2016)., who emphasized the role of education in preserving cognitive function despite increasing pathological burden. Similarly, Hoenig et al (Hoenig et al., 2017). reported that in the early phases of AD, higher educational attainment may contribute to the reversal of neurodegenerative effects and help sustain cognitive function—a hypothesis echoed in previous literature.

Collectively, these studies underscore the critical role of education in modulating cognitive resilience. While education does not appear to directly influence the degree of Alzheimer’s pathology, it significantly shapes the relationship between neuropathology, dementia status, and cognitive outcomes. The protective effects of education are most evident during the preclinical and early stages of disease progression. However, as pathology advances beyond a critical threshold, the benefits of education may wane. Intriguingly, some evidence suggests that in later stages, higher education could be associated with a steeper trajectory of cognitive decline, possibly due to greater cognitive reserve masking symptoms until pathology is more advanced. This aligns with the "cognitive reserve hypothesis," where individuals with higher education levels experience a delayed onset of cognitive decline, followed by a more rapid deterioration once decline begins (Scarmeas et al., 2006, Stern et al., 1995). Thus, while education may delay the clinical manifestation of dementia, it may also contribute to a sharper decline once compensatory mechanisms are overwhelmed.

The cognitive reserve hypothesis provides a foundational framework, proposing that individuals with greater educational attainment possess enhanced resilience to neuropathological damage through more efficient or flexible brain networks. However, beyond this hypothesis, other mechanisms such as increased synaptic density, greater neuroplasticity, and compensatory neural activity also play critical roles. For instance, higher education is associated with increased dendritic branching, synaptogenesis, and maintenance of gray matter volume, which may contribute to delayed clinical manifestation of cognitive impairment despite accumulating amyloid burden (Valenzuela and Sachdev, 2006). Functional imaging studies further support this, demonstrating that highly educated individuals engage alternative neural networks or more bilateral brain activation patterns during cognitive tasks—a phenomenon referred to as compensatory recruitment (Barulli and Stern, 2013).

Recent studies have employed stochastic change point models and multistate modeling to address this issue effectively (Hall et al., 2009, Yu et al., 2012). Additionally, there is evidence suggesting that education and cognitive training can delay the onset of significant cognitive decline (Muniz-Terrera et al., 2013). Numerous studies have also identified associations between cognitive and social experiences, along with related traits, and dementia risk. Clinical-pathological studies have consistently shown a weak correlation between the extent of brain damage and the presence or severity of dementia. This inconsistency suggests that other factors likely contribute to the preservation or decline of cognitive function. These factors, often referred to as neural or cognitive reserve, play a key role in maintaining cognitive function.

The observed association between higher educational attainment and resilience to Alzheimer’s pathology supports the cognitive reserve hypothesis and underscores the long-term benefits of early and sustained educational engagement. These insights have significant implications for public health: policies that promote equitable access to quality education from early life onward could serve as powerful preventive strategies against late-life cognitive decline. Moreover, lifelong learning initiatives and cognitive stimulation programs for older adults may enhance cognitive reserve and delay dementia onset, even in the presence of underlying neuropathology. Integrating these findings into public health planning could lead to cost-effective, scalable interventions with far-reaching impact on aging populations. Encouraging cognitive health through education-focused social policies aligns with a preventative model of care and addresses health disparities related to educational and socioeconomic inequalities (Livingston et al., 2020, Stern, 2012).

Furthermore, providing specific recommendations for future research is essential. Although this meta-analysis sheds light on the association between educational attainment and amyloid-beta accumulation across cognitive stages, several important gaps remain. One key limitation is the reliance on cross-sectional data, which restricts the ability to draw causal inferences regarding the temporal relationship between education, amyloid deposition, and cognitive decline. Longitudinal studies are urgently needed to track individuals from early adulthood into later life, capturing the timing and progression of neuropathological changes relative to educational exposure. Additionally, future research should explore how other dimensions of cognitive reserve—such as occupational complexity, lifelong learning, and social engagement—interact with education to influence disease trajectories. Incorporating multimodal biomarkers (e.g., tau, neurodegeneration markers, functional MRI) and genetic risk factors such as APOE ε4 status will be essential to disentangle the mechanisms through which education confers resilience or resistance. Finally, diverse population-based studies are needed to assess whether these associations hold across different sociocultural, racial, and socioeconomic contexts. Addressing these gaps will provide a more comprehensive understanding of the long-term protective effects of education and help inform personalized dementia prevention strategies.

While education may reflect broader socioeconomic factors that influence overall health, its positive impact on health may not be uniform across different levels of educational attainment. It is important to note, however, that this meta-analysis is based on a limited number of studies, which could introduce bias into the results. Therefore, future research, including larger-scale observational studies, is recommended to provide a more comprehensive understanding of the complex relationship between education, pathology, and cognition.

## Conclusion

This meta-analysis highlights a significant association between educational attainment and both amyloid accumulation in individuals with mild cognitive impairment (MCI) and the development of Alzheimer’s disease (AD). The findings suggest that higher levels of education may influence patterns of amyloid deposition and contribute to a protective effect against cognitive decline. These results are consistent with the cognitive reserve hypothesis, which posits that education strengthens the brain’s resilience to neuropathological damage, potentially delaying the onset of clinical symptoms.

Nevertheless, the observed heterogeneity across studies underscores the complexity of this relationship. Factors such as genetic predisposition, lifestyle behaviors, and regional or cultural differences may modulate the impact of education on neurodegeneration, warranting further investigation. While the inverse association between education and AD risk supports the notion that lifelong learning and cognitive stimulation may lower the likelihood of dementia, causality remains to be firmly established.

To advance our understanding, future research should prioritize longitudinal designs, include larger and more diverse cohorts, and adopt standardized methodologies. Investigating the effects of both early-life and lifelong educational experiences on neurobiological trajectories could yield critical insights into the mechanisms of cognitive resilience. Ultimately, this knowledge may inform the development of targeted, education-based interventions for dementia prevention and cognitive health promotion across the lifespan.

## Compliance with ethical standards

Not applicable.

## Funding

None

## Ethical statement

Not applicable

## CRediT authorship contribution statement

**Sayedeh-Fatemeh Sadat-Madani:** Writing – original draft. **Arimzadeh Omid K:** Writing – review & editing, Writing – original draft. **Niloofar Deravi:** Validation, Supervision, Project administration, Conceptualization. **Ata Akhtari kohnehshahri:** Writing – review & editing, Writing – original draft. **Parviz Aghaei Borzabad:** Supervision, Methodology, Investigation. **Mahya Ahmadpour Youshanlui:** Writing – review & editing, Writing – original draft. **Mahsa Asadi Anar:** Project administration, Methodology, Investigation. **Farzad Sheikhzadeh:** Writing – original draft, Data curation. **Mahsa Soleimanzadeh:** Writing – review & editing. **Elaheh Foroughi:** Writing – original draft, Data curation. **Ebrahimnia Yegane:** Writing – original draft. **Sedghi Fateme:** Writing – review & editing, Writing – original draft, Data curation. **Shaghayegh Afshari:** Writing – original draft. **Amirhesam Amirbeyk:** Writing – original draft. **Peyman Parhiz:** Writing – original draft. **Hani Ghadri:** Writing – original draft.

## Declaration of Competing Interest

The authors have no conflicts of interest to declare regarding the study described in this article and preparation of the article.
